# Phage Lytic Enzymes

**DOI:** 10.3390/v11020113

**Published:** 2019-01-29

**Authors:** Yves Briers

**Affiliations:** Department of Biotechnology, Ghent University, 9000 Ghent, Belgium; yves.briers@ugent.be

Phage lytic enzymes are enzymes produced by bacterial viruses, either as part of their virion to facilitate bacterial infection through local peptidoglycan degradation, or as soluble proteins to induce massive cell lysis at the end of the lytic replication cycle. In a pioneering paper from the Fischetti laboratory, the in vivo efficacy of streptococcal phage C_1_ lysin was demonstrated against group A streptococci in a mouse respiratory model [1]. In the following years, the field of phage lytic enzymes has drastically expanded, first for Gram-positive pathogens, and later also for Gram-negative pathogens through protein engineering [2,3]. Many new phage lytic enzymes active against diverse pathogens have been discovered and characterized. Their in vitro and in vivo properties as anti-infectives have been reported for an increasing number of applications or clinical indications. I am pleased to have witnessed the growing community of researchers focusing on phage lytic proteins during the last decade. In this issue, Vincent Fischetti provides an historical and personal perspective on the development of phage lytic enzymes as antimicrobials in his laboratory preceding this global attention for phage lytic enzymes [4].

From the growing body of preclinical evidence, phage lytic enzymes are now generally considered as a novel class of antimicrobials with great potential. Their rapid action, strong bactericidal effect, lack of resistance development and activity against persister cells are all related with their unique mode of action. Eighteen years after the first published in vivo efficacy tests, this special issue coincides with the first ongoing clinical phase II trials. The clinical evaluation of phage lytic enzymes has now caught up with the clinical evaluation of their parental producers; i.e., bacteriophages. The latter have been considered as antimicrobials since their discovery at the beginning of the 20th century, but were confronted with several technical and regulatory hurdles. Both phages and phage lytic enzymes represent novel classes of antimicrobials that are differentiated from the traditional small molecule antibiotics, responding to the urgent call of novel antibiotics [5].

This special issue provides an excellent review on the basic biology of phage lytic enzymes during both infection and lysis [6]. This review goes hand in hand with a review from the Azeredo group that focuses on the challenges and future prospects for in vivo therapy [7]. The authors emphasize the difficulties of translating the in vitro performance of phage lytic enzymes to in vivo efficacy and give recommendations to increase the success rate. One of them is the engineering of phage lytic enzymes by domain swapping. Two applications of a chimeric lysin, specifically ClyR which has extended antistreptococcal activity, have been reported in this special issue. Xu and colleagues demonstrated the anticaries efficacy of ClyR in a rat model [8], while Li and colleagues report the efficacy of ClyR against planktonic and sessile *Enterococcus faecalis* in an ex vivo dental model [9]. The antibiofilm activity appears to be again essential for success. From this perspective, the synergy between a depolymerase degrading extracellular polysaccharides and an endolysin has been studied in both static and dynamic biofilm models [10]. When bringing phage lytic enzymes closer to applications, formulation will be increasingly important. In this issue, the formulation of an anti-*Staphylococcus aureus* endolysin has been reported in an Aquaphor-based ointment along with the peptide apigenin for its anti-inflammatory and anti-haemolysis activity [11].

The discovery of new phage lytic enzymes remains essential to exploiting the best of the large natural diversity. Four new endolysins are reported and described in this special issue. Three *Bacillus* phage endolysins with broad antimicrobial activity against *Bacillus cereus sensu lato* strains were identified and characterized [12]. Melo and colleagues isolated an endolysin from a new *S. aureus* phage with antibiofilm activity [13]. Many *S. aureus* endolysins, including the one described by Melo in this issue, have a central amidase domain for which the function is still unknown. An auxiliary role of this amidase domain in the binding affinity of the cell wall binding domain is proposed, resulting in increased antimicrobial activity [14].

The majority of reports in this special issue are focused on phage lytic enzymes targeting Gram-positive pathogens, whereas the need for novel alternatives to treat Gram-negative infections is clinically the most pressing. The recurrent challenge in the design of phage lytic enzymes targeting Gram-negative pathogens is the outer membrane, requiring engineering or intrinsic outer membrane-permeabilizing activity. The study of Sykilinda and colleagues contributes to this field [15]. They present the three-dimensional structure of AcLys. The enzyme is featured by a highly charged C-terminal α-helix. This peptide is presumed to interact with and transiently permeabilize the outer membrane. Mycobacterium represents another group of bacteria with an urgent necessity for novel therapeutics, but which remains challenging for phage lytic enzymes because of the unusual outer membrane structure based on mycolic acids. Despite the large number of known phage lytic enzymes described in Mycobacteria phages [16], their complicated cell wall structure requires more extensive research to be overcome by phage lytic enzymes. In this issue, the current knowledge on Mycobacterium phages and their phage lytic enzymes, focused on the model endolysin from phage Ms2, and their applications as anti-infectives, has been reviewed [17].

Phage lytic enzymes should not only be considered for applications in health care. For virtually any situation in which bacteria are harmful, it becomes relevant to study phage lytic enzymes as a potential solution. For example, the bacterial contamination of yeast fermentations may decrease the yield and thus the revenues. The use of a yeast strain either producing intracellularly or secreting a lytic protein was studied to protect bioethanol fermentations from contamination with lactic acid bacteria, resulting in improved bioethanol productivity [18].

We hope the research articles and reviews of this special issue will provide further inspiration to expand phage lytic enzymes as a broad class of promising anti-infectives. While many beneficial properties of those lytic enzymes are broadly shared, the number of applications is *legio.* We wish to encourage all readers to continue the in-depth research of phage lytic enzymes, to initiate new applications and to further fill the clinical development pipeline.
